# Effects of N-acetylcysteine and glutathione ethyl ester drops on streptozotocin-induced diabetic cataract in rats

**Published:** 2008-05-12

**Authors:** Shu Zhang, Fei-Yan Chai, Hong Yan, Yong Guo, JJ Harding

**Affiliations:** 1 Department of Ophthalmology, Tangdu Hospital, Fourth Military Medical University, Xi’an, China; 2 Nuffield Laboratory of Ophthalmology, University of Oxford, Walton Street, Oxford, UK

## Abstract

**Purpose:**

To evaluate the effect of N-acetylcysteine (NAC) and glutathione ethyl ester (GSH-EE) eye drops on the progression of diabetic cataract formation induced by streptozotocin (STZ).

**Methods:**

One hundred and thirty Sprague-Dawley (SD) rats were selected, and diabetes was induced by streptozotocin (65 mg/kg bodyweight) in a single intraperitoneal injection. The control group (group I) received only vehicle. Then, 78 rats with random blood glucose above 14 mmol/l were divided into four groups (group II-V). The drug-treated rats received NAC and GSH-EE eye drops five days before STZ injection. Group I and V animals received sodium phosphate buffer drops (pH 7.4), and those in groups II, III, and IV received 0.01% NAC, 0.05% NAC, and 0.1% GSH-EE drops, respectively. Lens transparency was monitored with a slit lamp biomicroscope and classified into six stages. At the end of four weeks, eight weeks, and 13 weeks, animals were killed and components involved in the pathogenesis of diabetic cataract including thiols (from glutathione and protein), glutathione reductase (GR), catalase (CAT), and glycated proteins were investigated in the lens extracts. Blood glucose, urine glucose, and bodyweight were also determined.

**Results:**

The progression in lens opacity induced by diabetes showed a biphasic pattern in which an initial slow increase in the first seven weeks after STZ injection was followed by a rapid increase in the next six weeks. The progression of lens opacity in the treated groups (group II-IV) was slower than that of the untreated group (group V) in the earlier period and especially in the fourth week. There were statistically significant differences between the treated groups and the untreated group (p<0.05). However, these differences became insignificant after the sixth week, and the progression of lens opacification in all diabetic groups became aggravated. The content of thiol (from glutathione and protein), glutathione reductase (GR), and catalase (CAT) were lower in the lens extracts of the diabetic rats four weeks, eight weeks, and 13 weeks after the STZ injection while the levels of thiol and CAT activity were both higher in the treated groups (group II-IV) than in the untreated group (group V) at every stage. However, there was no statistically significant difference (p>0.05). Moreover, the diabetes resulted in an increased level of glycated proteins in both the treated groups and the untreated group, but there was no statistically significant difference between all the diabetic groups (p>0.05).

**Conclusions:**

NAC and GSH-EE can slightly inhibit the progression of the diabetic cataract at the earlier stage. They may maintain lens transparency and function by serving as a precursor for glutathione biosynthesis and by protecting sulfhydryl groups from oxidation.

## Introduction

Understanding the mechanisms of the development of cataract and looking for available therapeutic methods are problems of scientific and social interest because cataract is one of the major causes of reversible blindness. It is estimated that over 50 million people worldwide suffer from cataracts, and the problem will grow in parallel with aging of the population [1,2]. At present, the cure for cataract is still surgery. However, surgery is not equally available to all, and where it is available, it does not produce equal outcomes [2,3]. In addition, risk and cost factors also drive the study of pharmaceutical approaches to the maintenance of lens transparency.

The role of oxidative stress in diseases including diabetes and the many changes in the development of cataracts is controversial [4,5]. The reducing compound glutathione (GSH), quantitatively the most important endogenous rechargeable antioxidant, exists in an unusually high concentration in the lens where it functions as an essential antioxidant vital for maintenance of the tissue’s transparency [6-8]. The GSH system plays a key role in the protection against oxidative stress. Depletion of GSH is found in many cataractous lenses [1,4,7,9,10]. The depletion of GSH or the inhibition of the redox cycle allows low levels of oxidant to damage lens epithelial targets such as Na/K-ATPase, certain cytoskeletal proteins, and proteins associated with normal membrane permeability. In addition, GSH has several important functions related to amino acid transport across membranes, protein synthesis and degradation, gene regulation, and cellular redox regulation [7,11,12]. Therefore, supplementation of GSH to the lens may help to maintain its protective ability against oxidative stress and other attacks and lead to a slower age-related loss of antioxidant activity of lens and eventually to delay the onset of cataract [3].

Since by itself GSH is not effectively transported into cells, several alternatives to increase intracellular levels of GSH have been developed [12-17]. These alternatives include N-acetylcysteine (NAC) and GSH esters to boost GSH in the cell [18]. GSH esters are hydrolyzed to GSH in cells. NAC is hydrolyzed to cysteine in the cell. It is the availability of cysteine that usually limits GSH synthesis. NAC limits protein carbonyl formation in incubated rabbit lens epithelium [19]. NAC, GSH monoester, and glutathione isopropyl ester injected intraperitoneally were effective in delaying or preventing cataract formation induced by X-irradiation or buthionine sulphoxide [14,15]. NAC can combine with other anti-cataract drugs to prevent the progression of lens opacification [20,21]. The potential benefit of supplementation with NAC and/or GSH ester in the prevention of the development of cataract among the diabetic population is clear [19].

GSH esters have shown considerable merit following the inhibition of GSH synthesis and in the reversal of low GSH in several pathological and clinical conditions requiring high levels of tissue GSH [22]. The ester is converted intracellularly into GSH [13]. GSH ester can prevent buthionine sulfoximine-induced cataracts and lens epithelial cell damage [14]. Moreover, γ-glutamylcysteine ethyl ester is able to inhibit L-buthionine sulfoximine-induced cataract formation by mitigating the deprivation of glutathione and by elevating the level of glutathione in cultured lens [23]. GSH ethyl ester (GSH-EE) supplementation of mice improved endurance performance and prevented muscle lipid peroxidation by altering glutathione homeostasis during prolonged exercise [16]. The antioxidative effect of NAC and GSH esters had been demonstrated from many studies in vivo [24,25] and in vitro [17,26]. They made great contributions to research on the biologic effects and medical application of NAC and GSH esters.

However, the researches above were performed with difficulty for the drugs were administrated orally [27] or intraperitoneally [28], and there were many complications [18,29]. If a drug therapy is to be developed for cataract, it must be extremely safe because a successful surgical procedure is already available. Use of eye drops would avoid potential systemic complications. The streptozotocin (STZ)-induced cataract model in rats has been widely used successfully for cataract studies [30,31]. We previously used the streptozotocin (STZ)-induced cataract model in rats successfully to assess aspirin, paracetamol, and ibuprofen as anti-cataract drugs [32]. In the present study, NAC and GSH-EE eye drops were used for the first time in a STZ-induced cataract study. The aim of the present study was to investigate the effect of treatment with NAC and GSH-EE eye drops on the development of streptozotocin-induced cataract in diabetic rats.

## Methods

### Materials

Streptozotocin (STZ), N-acetylcysteine (NAC), glutathione ethyl ester (GSH-EE), and 5-hydroxymethylfurfural (5-HMF) were purchased from Sigma Chemical Company (Beijing, China). Sprague-Dawley rats were provided by Animal Laboratories of Fourth Military Medical University (Xi’an, China). Protein and enzyme quantification kits were obtained from Jiancheng Biology Company (Nanjing, China). Glucotrend 2 was from Roche Diagnostic Limited Company (Xi’an, China). Tes-Tape was from Zhujiang Biochemistry Reagents (Guangzhou, China). All other chemicals and solvents were of analytical grade and were obtained from local companies.

### Experimental design

The experiments lasting over 13 weeks were performed using Sprague-Dawley rats (obtained from the Laboratory Animal Research Centre, Fourth Military Medical University, Xi’an, China), which were housed in individual polypropylene breeding cages under a day/night cycle of 12 h at 20–25 °C room temperature. All animals had unlimited access to water. Animal care and protocols were in accordance with and approved by the Institutional Animals Ethics Committee and conformed to the ARVO Statement for the Use of Animals in Ophthalmic and Vision Research.

All the lenses were examined by slit lamp microscopy before induction of diabetes, and those with any defect of lens or cornea were rejected. Ten rats were randomly selected for the normal group (group I), and 120 others were single injected intraperitoneally, avoiding the intestine, with STZ (65 mg/kg bodyweight), which was dissolved in 20 mM sodium citrate buffer, pH 4.5 (10 mg STZ/ml citrate buffer) after the pH value was adjusted to 7.4. The STZ solution was sterilized through a 0.22 µM Millipore filter into a sterilized container kept on ice and used within 10 min of dissolving. The normal control group (group I), the non-diabetic rats, were injected with a sterilized buffer. After three days the blood of each rat was tested for glucose by using Glucotrend (Roche Company, Germany). The urine of each rat was tested for glucose with Tes-Tape at the same time. Any STZ-injected rat that had no detectable glucose in the urine was rejected (about 35% of those injected). Fasting blood glucose was then measured in all rats to confirm that the streptozotocin-injected rats were diabetic (more than 14 mmol/l) and that the control rats were not. Then, the diabetic rats were divided into four groups. Seventy-eight diabetic rats were divided into four groups (group II-V) and received 0.01% NAC drops (group II; n=19), 0.05% NAC drops (group III; n=19), 0.1% GSH-EE drops (group IV; n=19). The untreated diabetic group constituted group V (n=21). The rats were weighed, and five groups (group I-V) were established with a comparable weight distribution (ranging from 130.0±27.24 g to 142.8±27.91 g; p>0.05). There was no significant difference in weight among these groups.

### Preparation of eye drops

The drugs (0.01 g and 0.05 g of NAC) were dissolved in 100 ml of sodium phosphate buffer (pH 7.4). After vibration, drugs were completely dissolved with the pH value of 0.01 g and 0.05 g of NAC drops were 7.4 and 7.1, respectively. Five milligrams of GSH-EE was dissolved in 5 ml of sodium phosphate buffer solution (pH 7.4). The pH value of the GSH-EE solution was adjusted to 7.2 before use.

Five days before the rats were injected with STZ, all rats except the normal group were treated with NAC or GSH-EE drops twice daily. The eye drops were tested for safety and utility by preliminary experiments. There was no significant statistical difference between the eyes of drug-treated rats and untreated rats when observed for irritation and conjunctiva congestion. The drug solutions were made fresh daily and preserved in a 4 °C icebox. During dropping, the rats were held by assistants, and their eyelids were held open. Drops, two at a time, were administered, and rats were continuously held for another 2–3 min with light massage of the eyeball to sustain the volume dose. Initially, the rats were sensitive to this dropping but became calm after few days of experience. The rats were fed with standard chow ad libitum.

### Slit lamp examination and cataract classification

**Figure 1:**
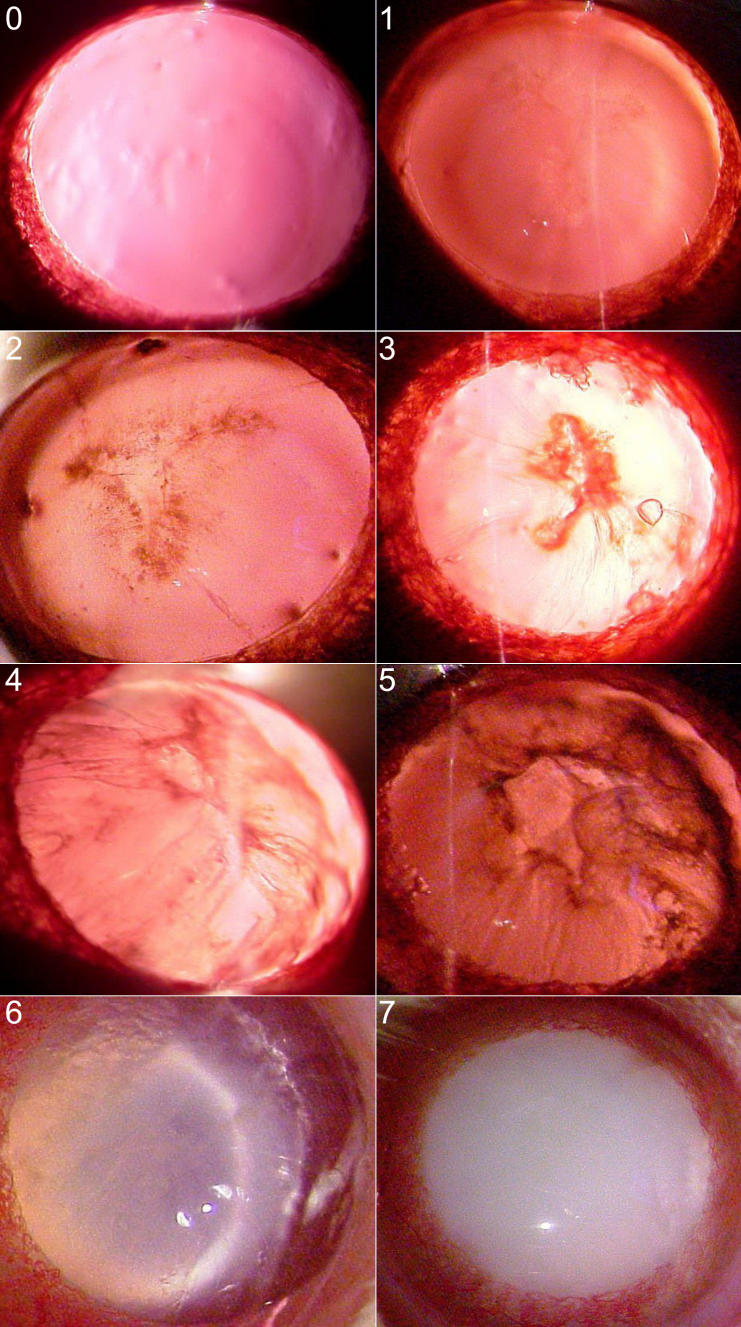
The photograph of all grading lens at the slit lamp (Haag-Streit BQ 900). The grades are as follows: grade 0, clear; grade 1, clear nuclear with wide sutures; grade 2, slight dense nuclear with opacities radiating from sutures; grade 3, dense nuclear without clefts; grade 4, dense nuclear with clefts; grade 5, nuclear cataract with clefts; grade 6, nuclear cataract with dense radial opacities; and grade 7, nuclear cataract with whole lens opacities.

Eyes were examined every week using a slit lamp biomicroscope (Haag-Streit BQ 900 model; Hagg-Streit International, Koeniz, Switzerland) on dilated pupils. Initiation and progression of lenticular opacity was assessed according to the Oxford system [32]: grade 0, clear; grade 1, clear nuclear with wide sutures; grade 2, slight dense nuclear with opacities radiating from sutures; grade 3, dense nuclear without clefts; grade 4, dense nuclear with clefts; grade 5, nuclear cataract with clefts; grade 6, nuclear cataract with dense radial opacities; and grade 7, nuclear cataract with whole lens opacities (Figure 1). The stage of cataract was scored according to the classification described above.

### Lens preparation

On the 4^th^, 8^th^, and 13^th^ week, animals selected randomly from each group were sacrificed by decapitation and their eyeballs were removed for biochemical evaluation. Eyeballs were soaked in 0.9% neutral normal saline, and the lenses were dissected by the posterior approach then placed into pre-weighed Eppendorf tubes and frozen at –40 °C until further analysis. On the fourth and eighth week, 4, 6, 6, 6, and 6 lenses were used in groups I, II, III, IV and V, respectively, as well as 10, 12, 10, 12, and 10 lenses on the 13th week, respectively.

### Protein determination

Protein concentration was determined by Coomassie brilliant blue method using protein assay kit from Jiancheng Company (Nanjing, China).

All lenses were ground in 0.9% neutral normal saline (1:19) and homogenized and then centrifuged in Eppendorf tubes. A clear supernatant was used for protein determination, which was according to the method described with the kits.

### Thiol determination

Thiols were measured using the dithio-bis-nitrobenzoic acid (DTNB) method at 25 °C and 412 nm [33,34]. The clear supernatant liquid used for protein determination was taken by suction from the centrifuged Eppendorf tube, and the thiol content was determined according to the description of the kit. Thiols react with dithio-bis-nitrobenzoic acid to give a yellow compound that has a high absorption of light at 412 nm. This absorption was measured. Through this colorimetric method, the content of thiol in each lens (from GSH and protein) was measured.

### Assay of glutathione reductase activity

Glutathione reductase (GR) activity was measured according to the procedure of Linetsky et al. [35]. The reaction was initiated by the addition of 20 μl of lens homogenate. Oxidized glutathione (GSSG) was reduced to GSH catalyzed by GR with NADPH as a cofactor. The decrease in the optical density at 340 nm was recorded at 25 °C for 2 min. The units of enzymatic activity were calculated using an extinction coefficient of 6.22 mM/cm for NADPH. One unit was equivalent to the oxidation of 1 mmol of NADPH per min.

### Assay of catalase activity

Catalase (CAT) activity in the lens was assayed with hydrogen peroxide as the substrate using a method based on the direct measurement of H_2_O_2_ decomposition [36]. The final volume of each enzyme assay was 3 ml of substrate and 20 μl of the supernatant of lens homogenate. The assay was performed at 25 °C and at 240 nm. Enzyme activity was expressed as units per gram of protein, and one unit of CAT activity represented 1 mmol H_2_O_2_ decomposed per min.

### Assay of glycation of lens proteins

The determination of the amount of glycation of lens proteins was based on the thiobarbituric acid method [37]. The 5-hydroxymethylfurfural (5-HMF) was released into solution on boiling glycated protein in the presence of a weak acid. Any solubilized protein was then precipitated out of solution by centrifugation. Thiobarbituric acid forms an adduct with 5-HMF that absorbs at 443 nm. The value was expressed as units (mmoles HMF per mole protein).

### Statistical analysis

One-way ANOVA was used for testing statistical significance between groups. The median calculation of the lens opacity for each group was analyzed by using the Wilcoxon rank sum test. p<0.05 was considered significant. All the data were dealt by the SPSS 11.0 statistical package.

## Results

### Blood glucose after injection of streptozotocin

Ten rats were selected for the normal group, and the others (120 rats) were injected with STZ. Based on the level of plasma glucose, the 120 rats were designated as non-diabetic rats (<14 mmol/l plasma glucose) and diabetic rats (>14 mmol/l plasma glucose).

Based on the monitoring of blood glucose over 72 h, we found that random blood glucose testing was more reliable to identify diabetic rats than fasting blood glucose measurement. About 65.0% (78/120) of the rats responded to the STZ injection (blood glucose>14 mmol/l) according to the random blood glucose testing. Two weeks after the rats were made diabetic, three rats were rejected because the blood glucose had fallen below 14 mmol/l (one rat was from group II and two rats were from group V). Therefore, all the data of these rats were excluded from the statistical analysis. Then, 75 rats in the treated groups were used for statistical analysis.

The diabetic rats had a much higher blood glucose level than the control rats throughout the experimental period (Table 1). There was no significant difference in blood glucose levels between the four groups of diabetic rats (p>0.05; Table 1). Treatment with either NAC or GSH-EE did not reverse the changes in blood glucose, showing that the NAC and GSH-EE treatment had no effect on hyperglycemia.

**Table 1:** The changes of blood glucose after injection of streptozotocin.

**Group**	**n**	**72 h**	**4 weeks**	**8 weeks**	**13 weeks**	**p**
I	10	6.99±0.42	7.01±0.46	6.13±0.35	6.20±0.73	0.470
II	18	29.18±5.80a	28.73±3.23a	31.46±4.32a	29.39±9.16a	0.341
III	19	28.94±5.53a	27.51±3.01a	31.90±2.20a	31.76±1.69a	0.375
IV	19	28.13±2.68a	29.12±3.05a	31.93±1.99a	31.20±1.87a	0.251
V	19	29.43±5.23a	28.61±5.68a	32.00±1.41a	29.48±7.63a	0.840

Each value is the mean±SD of results in all the animals in a given group. “a” denotes that p<0.05 when comparing the normal group (group I) with the four diabetic groups (group II-V). There were no statistically significant differences between the four diabetic groups. In the column headed “p” the value represents the comparison between four different times. Group I: normal; II: NAC-treated (0.1 mg/ml); III: NAC-treated (0.5 mg/ml); IV: GSH-EE-treated (1 mg/ml); V: untreated diabetic.

### The changes of rat’s weights

**Figure 2:**
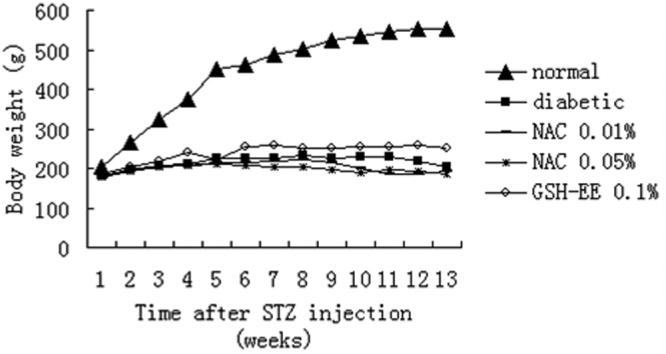
Changes of rat’s bodyweight before and after the injection of streptozotocin. Data are the average of results in all the animals in a given group. The weights of the normal rats were significantly different from the four diabetic groups (p<0.05), and there were no significant differences between the four diabetic groups (p>0.05) at any time.

In all five groups, weight was gained in a biphasic manner, an initial steep increase for the first six weeks, followed by a slower change during the next seven weeks (Figure 2). The diabetic rats had lower weight gain compared with the normal rats during the experimental period (p<0.05; Figure 2). In the normal group, the bodyweight increased quickly. On the other hand, there were only slight increases in the other four diabetic groups with a plateaued weight of about 200 g. There was no statistically significant difference in the weight gain among the groups of diabetic rats (p>0.05; Figure 2).

### Grading of lens opacification

The onset of cataract was observed after two weeks by slit lamp examination. The median calculation of lens opacity was presented in Table 2. All the lenses in group I appeared to be clear and normal throughout the experimental period whereas after four weeks, only 30% of the lenses were in grade 1, 60% in grade 2, 10% in grade 3 of cataract formation, and none of them were clear (grade 0; Table 2) in group V, the untreated diabetic rats. However, on the fourth week, more than 50% of the lenses were clear in groups II and IV whereas in group V, most of the lenses were in grade 2 and only a few were clear (p<0.05; Table 2). After five weeks, lens opacification became worse in groups II-V with no significant difference between the diabetic groups, treated and untreated (p>0.05; Table 2). These observations indicate that NAC drops and GSH-EE drops delayed progression of hyperglycemia-induced cataract at the earlier period. At the end of 11 weeks, most of the lenses (65%) in groups II-V showed development of mature cataract (grade 7: nuclear cataract with whole lens opacities; Table 2).

**Table 2:** The effect of NAC and GSH-EE on streptozotocin-induced lens opacity.

**Group**	**3 week**	**4 week**	**5 week**	**6 week**	**7 week**	**8 week**	**9 week**	**10 week**	**11 week**	**12 week**	**13 week**
I	0	0	0	0	0	0	0	0	0	0	0
II	0	0*	2	2	3	4	5	6	7	7	7
III	0	1	2	3	4	4	5	6	6	7	7
IV	0	0^#^	2	2	4	4	5	5	6	7	7
V	1	2	2	3	4	5	5	6	6	7	7

Values are expressed as medians. Median representation: 0, clear; 1, clear nuclear with wide sutures; 2, slight dense nuclear with opacities radiating from sutures; 3, dense nuclear without clefts; 4, dense nuclear with clefts; 5, nuclear cataract with clefts; 6, nuclear cataract with dense radial opacities; and 7, nuclear cataract with whole lens opacities. Statistical analyses were performed by using Wilcoxon rank sum test. Note: The asterisk denotes that p=0.023 (group II compared with group V on the fourth week). The asterisk denotes that p=0.031 (group IV compared with group V on the fourth week). Group I: normal; II: NAC-treated (0.1 mg/ml); III: NAC-treated (0.5 mg/ml); IV: GSH-EE-treated (1 mg/ml); V: untreated diabetic.

Remarkably, the grade of lens opacification in groups II and IV almost stay clear before four weeks while in group V, the lens opacification progressed almost to grade 2 after three weeks (Table 2). This observation indicates that NAC and GSH-EE can delay the onset of diabetic cataract, but they failed to delay or reverse the severity of cataract at later stages.

### Activity of enzymes

The water-soluble protein concentration was unchanged in various grades of lens opacification (Table 3). A decrease of thiol content was found in the lens of diabetic rats (groups II-V) at different stages after STZ injection (p<0.05; Table 4). The level of thiols (from GSH and protein) in the lens of diabetic rats decreased to about 50% of the level of the normal controls at each different stage while the content of thiol slightly decreased with time in the normal group. Surprisingly, NAC and GSH-EE enhanced thiol levels by about 12%–18% compared to the untreated group on the fourth week (Table 4), but it was statistically insignificant. The activity of GR decreased by 20% in the diabetic rats at all stages, but this difference was not statistically significant. No change of GR activity was observed during cataract progression in the diabetic rats (Table 5).

**Table 3:** The water-soluble protein in various grade of lens opacification.

**Time**	**Group I**	**Group II**	**Group III**	**Group IV**	**Group V**
4th week	5.29±0.5	4.35±0.5	4.32±0.9	5.14±0.4	4.99±0.5
8th week	5.03±0.3	4.01±0.5	4.12±0.9	4.36±0.3	4.40±0.3
13th week	4.73±0.5	4.37±0.7	4.30±0.8	4.21±0.6	4.33±0.8

The data are the mean±SD (group I, n=6 lenses; other groups, n=12 lenses at each time). The water-soluble protein is expressed as g/l. There were no significant differences during all the five groups at various grade of lens opacification. Group I: normal; II: NAC-treated (0.1 mg/ml); III: NAC-treated (0.5 mg/ml); IV: GSH-EE-treated (1 mg/ml); V: untreated diabetic.

**Table 4:** The effect of NAC and GSH-EE on the thiol content of rat lenses.

**Time**	**Group I**	**Group II**	**Group III**	**Group IV**	**Group V**
4th week	118.2±22.04	80.50±21.21	78.31±19.10	76.32±20.02	68.44±23.45
8th week	115.1±19.57	52.50±20.11	54.43±19.60	52.62±22.82	50.27±24.18
13th week	105.68±19.06	40.50±19.07	39.59±21.61	40.60±20.12	40.54±18.17

The data are the mean±SD (group I, n=6 lenses; other groups, n=12 lenses at each time). The total thiol is expressed as milligrams per gram protein. No significant differences were observed among the four diabetic groups (group II-V) at any given stage. Group I: normal; II: NAC-treated (0.1 mg/ml); III: NAC-treated (0.5 mg/ml); IV: GSH-EE-treated (1 mg/ml); V: untreated diabetic.

**Table 5:** The effect of NAC and GSH-EE on the activity of GR in rat lenses.

**Time**	**Group I**	**Group II**	**Group III**	**Group IV**	**Group V**
4th week	6.76±2.60	6.01±2.75	5.86±2.65	6.02±2.52	5.41±2.58
8th week	6.70±0.70	5.31±2.37	5.77±2.94	5.65±3.31	5.29±2.85
13th week	6.49±2.44	5.01±2.13	5.20±2.52	5.29±2.73	5.11±2.72

The data are the mean±SD (group I, n=6 lenses; other groups, n=12 lenses at each time). The activity of GR is expressed as μmol per gram protein. At all stages, the mean values for all diabetic groups were less than the normal group, but there were no significant differences between all five groups at various stages. Group I: normal; II: NAC-treated (0.1 mg/ml); III: NAC-treated (0.5 mg/ml); IV: GSH-EE-treated (1 mg/ml); V: untreated diabetic.

The activity of CAT was significantly lower in diabetic rats than in the normal rats (p<0.05; Table 6). In the present study, we observed a loss of approximately 40% of lens CAT activity in diabetic rats on the fourth week, 42% on the eighth week, and 55% on 13^th^ week (p<0.05). NAC and GSH-EE treatment resulted in a slight increase in CAT activity at the earlier cataract stages (Table 6).

**Table 6:** The effect of NAC and GSH-EE on the activity of CAT in rat lenses.

**Time**	**Group I**	**Group II**	**Group III**	**Group IV**	**Group V**
4th week	55.58±24.22	36.56±7.90	37.40±5.28	34.80±13.63	33.02±7.45
8th week	50.24±26.34	29.51±14.90	30.07±7.28	29.73±6.35	29.04±10.36
13th week	46.29±11.32	21.51±7.92	23.07±13.35	22.79±9.37	20.55±10.37

The data are the mean±SD (group I, n=6 lenses; other groups, n=12 lenses at each time). The activity of CAT is expressed as μmol per gram protein. There were no significant differences between the four diabetic groups (group II-V) at any given stage. A statistically significant difference was observed between the normal group (group I) and the untreated group (group V) in various stages (p<0.05). Group I: normal; II: NAC-treated (0.1 mg/ml); III: NAC-treated (0.5 mg/ml); IV: GSH-EE-treated (1mg/ml); V: untreated diabetic.

A rise of protein glycation was found in diabetic groups with statistically significant differences (p<0.05; Table 7). No effects of NAC and GSH-EE drops on the level of glycation were noted (Table 7).

**Table 7:** The effect of NAC and GSH-EE on the content of glycation in rat lenses.

**Time**	**Group I**	**Group II**	**Group III**	**Group IV**	**Group V**
4th week	4.10±0.21	4.80±0.34	4.77±0.25	4.81±0.23	4.89±0.29
8th week	4.33±0.29	5.61±0.34	5.00±0.25	5.22±0.25	5.25±0.28
13th week	4.87±0.23	5.77±0.37	5.91±0.30	5.82±0.27	5.90±0.31

The data are the mean±SD (group I, n=6 lenses; other groups, n=12 lenses at each time). The content of glycation is expressed as mmoles HMF per mole protein. There were no significant differences between the four diabetic groups (group II-V) at any given stage. The statistically significant difference was observed between the normal group (group I) and the untreated group (group V) in various stages (p<0.05). Group I: normal; II: NAC-treated (0.1 mg/ml); III: NAC-treated (0.5 mg/ml); IV: GSH-EE-treated (1 mg/ml); V: untreated diabetic.

## Discussion

Diabetic lenses in both humans and hyperglycemic animals were shown to be the subject of elevated oxidative stress [5,35,38]. Many drugs have been used for anticataract research in animals [1,14,39], and some have proved effective in the prevention of lens opacity. GSH is important to maintain lens proteins in a reduced state, and a healthy lens utilizes its various antioxidants and oxidation defense enzymes to protect itself against oxidation [1,40,41].

GSH itself is not membrane permeable thus it is not effectively transported into cells [42]. The NAC and GSH ester have acted as GSH precursors to regulate the content of GSH in tissues and cells [43,44]. NAC and GSH esters provide the direct and convenient means available for increasing the intracellular GSH concentration of many tissues and cell types [13,22].

In the present study, NAC and GSH-EE drops were used for the first time in STZ-induced diabetic cataract and showed an inhibition of early cataract. The drugs for NAC and GSH-EE are liposoluble substrates, which can permeate across biomembranes [45,46]. The administration route of previous studies in vivo and in vitro were performed intraperitoneally or taken orally, and both of these were performed with some complications [18,27-29]. This study investigated the feasibility and the efficacy of NAC and GSH-EE drops in diabetic cataract. During the whole process, there were no remarkable stimulating reactions on the eyes. The drops are proved to be better modalities than oral treatment for safety and convenience. The present concentrations of eyes drops were based on previous studies [15,28,47]. According to our previous studies, we chose the dose of eye drops consistent with the injected dose, and the volume dose was increased along with the increase of rats’ bodyweight. To overcome the limitation of the volume applied by a single dosage to rat eyes, we increased the frequency of administration to sustain the volume dose. Therefore, we made 0.1 mg/ml (0.01%) and 0.5 mg/ml (0.05%) NAC drop solutions and applied them to the rats’ eyes three times a day, and we made 1 mg/ml (0.1%) GSH-EE drops solution and applied them twice daily.

NAC and GSH-EE slightly inhibited the progression of diabetic cataract at the earlier stages. Slit lamp evaluation revealed the prevention of lens opacification, even though this function was statistically significant only at a very early stage (the fourth week after injection). However, there was no statistically significant difference in the progression of lens opacification between the two drugs at later stages or in the two different concentrations of the NAC-treated groups.

NAC and GSH-EE appeared to enhance the content of thiol (from GSH and protein) in the treated groups compared with the untreated group at every stage, but the differences were statistically insignificant. The difference in thiol content between the treated groups and the untreated group is more obvious in the fourth week than in the 8^th^ and 13^th^ week. This may be due to the GSH supplement being unable to cope with the large changes in mature cataract lenses. Among the benefits of GSH, it can decrease the rate of glycation [48].

NAC and GSH-EE did not prevent GR and CAT from decreasing in the treated groups. This may be due to permeability and an inability to achieve effective drug concentrations, the level of blood glucose being too high for NAC and GSH-EE against diabetic cataract, or the fact that the inactivation of these enzymes is not related to the concentration of GSH. Consistent with the results of others [49], the activity of GR was relatively unchanged during cataract progression in the diabetic rats. Glucose binds non-enzymically to this enzyme and inhibits it [37]. The activity of CAT in the lens may similarly be affected, resulting in a decrease in GSH. The early loss of GSH in diabetic cataract may result from the inactivation of various enzymes including CAT and GR, which have a role in maintaining GSH levels. In hyperglycemia, the levels of reactive oxygen species (ROS) in the lens rise. Enzymes that protect against oxidation may be inactivated and rendered ineffective by either glycation or oxidation. The data indicates that the beneficial effects of NAC and GSH-EE do not seem to be associated with GR and CAT activities.

Our results demonstrated an increase in glycation levels in addition to a decrease in certain antioxidant enzyme activities. This suggests NAC and GSH-EE as antioxidants are ineffective in inhibiting glycation at such high levels of hyperglycemia. It has been suggested that glycation of lens crystallins may cause conformational changes that result in the exposure of thiol groups to oxidation and cross-link formation [1,38,50]. Glycation, which causes the aggregation of lens crystallins that produce the high molecular weight material responsible for opacification, was demonstrated by animal studies [38,51].

The biochemical and morphologic changes in the NAC-treated groups were similar to the NAC-treated groups with two different concentrations were similar to the changes in the GSH-EE-treated group. The two drugs, administered before the STZ injection, resulted in a significant prevention of lens opacification at an early stage. NAC and GSH-EE as thiol-containing antioxidants can penetrate freely into the lens, increasing the level of GSH [17], and having benefits attributed to the participation of the SH group of GSH in the diabetic lens which inhibits oxidative stress in the lens [39]. There was no obvious dose-dependent effect in the two NAC-treated groups, which may be due to the similarity of the two concentrations or the limitations in penetrating into the lens.

In conclusion, the study showed NAC and GSH-EE delay the progression of lens opacification in diabetic rats in the earlier stage. They may serve as a precursor for glutathione biosynthesis, increase the levels of GSH, inhibit oxidative insult of crystallins and antioxidant enzymes, and thus protect the lens transparency. Therefore, research on the mechanism of NAC and GSH-EE provides an important experimental base for the drug treatment of cataract.

It remains to be determined whether NAC and GSH esters are limited in their transport into the eyes. These derivatives of GSH also need to be investigated with respect to their transport properties. The present and previous findings, which support the view that cataracts are produced by oxidation and glycation of lens proteins, suggest that procedures that increase lens GSH levels would protect against the development of cataract.
